# Highlights for Management of a Child with a Urinary Tract Infection

**DOI:** 10.1155/2012/943653

**Published:** 2012-07-19

**Authors:** Sabeen Habib

**Affiliations:** Department of Pediatrics, LSUHealth Shreveport, Shreveport, LA 71130, USA

## Abstract

Urinary tract infections remain the most common bacterial infection in childhood. *Escherichia coli* is responsible for over 80% of Pediatric UTIs. Other common gram negative organisms include Kleibsiella, Proteus, Enterobacter and occasionally Pseudomonas. Signs and symptoms vary greatly by age of the patient becoming more specific as the child grows older. Even in the absence of specific signs a UTI should be included in the differential diagnosis of high grade fever. In younger children, presence of upper respiratory infections, otitis media or gastroenteritis does not eliminate the possibility of a UTI. Culture of the urine remains the gold standard for diagnosing UTIs. All males and females with well documented UTIs should be imaged for the presence of urological anomalies associated with UTI. Depending on patient's clinical symptoms and tolerance, therapy can be oral or parenteral as they have both been found equally efficacious. Healthcare professionals should ensure that when a child or young person has been identified as having a suspected UTI, they and their parents are given information about the need for treatment, the importance of completing any course of treatment and advice about prevention and possible long-term management.

## 1. Overview

Urinary tract infections (UTIs) remain the most common bacterial infection in childhood [1]. The cumulative incidence of UTI in children by 6 years of age is 3%–7% in girls and 1%-2% in boys. This amounts to between 70 000 and 180 000 children in the United States developing UTI annually [1]. While most UTI is caused by bacteria, other infectious agents can cause UTI. These include viruses, fungi, and mycobacterial infections. Frequent urinary tract infections can result in chronic kidney disease and hypertension [2, 3].

## 2. Pathophysiology

In healthy children, urine in the collecting system and urinary bladder is sterile. The urethra on the other hand is colonized with bacteria. Urinary malformation, urine stasis, and adherence of bacteria to the uroepithelial mucosa are the main predisposing factors for the development of UTI. Congenital obstructive uropathy is often associated with UTI. The pathogenesis of UTI in detrusor sphincter dyssynergia syndrome is due to infrequent bladder emptying and stasis. This later condition sometimes also referred to as dysfunctional voiding [4]. Most bacterial urinary tract infections are ascending. Urogenital bacteria are often the most common causative agents. When stasis of urine is present, bacteria multiply and UTI can develop. 

## 3. Epidemiology

Most of the studies evaluating UTI in children are observational, hence conclusions from such studies are limited [5]. 

In males, it is more common during neonatal period and early infancy and it declines afterwards [6]. Usually associated with anatomical abnormalities and outlet obstruction. About 8% of girls (3% prepubertal), and 2% of boys (1% prepubertal) experience at least one episode of UTI up to the age of 7 [7]. It occurs in 0.1–0.4% of infant girls and increase up to 1.4% during 1–5 years and 0.7–2.3% in school age. The incidence is greater in girls in this age group and is likely due to short urethra and translocation of fecal bacteria. Close to 0.2% of circumcised and 0.7% of uncircumcised infant boys are at risk, which reaches to 0.1-0.2 during 1–5 years and 0.04–0.2 in school age [8]. UTI may lead to transient renal damage in 40% and permanent renal scarring in 5% of patients [9]. 

A multicenter study in 2007 revealed that the cumulative risk of UTI in children under age 6 years is 6.2% [10]. In older children with urinary symptoms with or without fever, the prevalence of UTI was 7.8% [11].

Asymptomatic bacteriuria occurs in 1% and 3% of infants and preschool age children, in about 1% of older children [12]. 

## 4. Etiology

*Escherichia coli* is responsible for over 80% of pediatric UTIs [5]. Other common gram negative organisms include Kleibsiella, Proteus, Enterobacter, and occasionally Pseudomonas [13]. *Proteus mirabilis* is a common pathogen in males and in children with kidney stones [8]. Gram-positive pathogens include group B Streptococcus and Enterococcus in neonates and infants, and *Staphlococcus saprophyticus* in adolescent girls [14]. Fungal infections are much less common and are usually to those who are immune-compromised or diabetic, are on long-term antibiotics, or have long-term indwelling catheter [5, 15]. Often urine is contaminated by Lactobacillus species, Corynebacterium spp., coagulase-negative staphylococci, and *α* hemolytic streptococci [5].

## 5. Clinical Presentation

### 5.1. History and Physical Examination

Signs and symptoms vary greatly by age of the patient becoming more specific as the child grows older. Even in the absence of specific signs, a UTI should be included in the differential diagnosis of high-grade fever. Asymptomatic bacteriuria is present in about 3% of preschool age children, as mentioned in the previous section. About a third of these patients will have some symptoms of urinary tract eventually.

In young infants, symptoms are usually nonspecific and may include lethargy, decreased feeding, increased sleep, vomiting, and decreased urinary output [16, 17]. Occult UTI in neonates can be presented with late-onset jaundice especially if conjugated fraction is elevated too [18].

In younger children, presence of upper respiratory infections, otitis media, or gastroenteritis does not eliminate the possibility of a UTI [19, 20]. In one study of febrile infants, those testing negative for RSV also had a positive urine culture 10.1% of the time, whereas those that tested positive for RSV had a positive urine culture 5.4% of the time [21]. Even the presence of varicella, herpangina, croup has been found to decrease the risk of UTI by 2.6% [5, 21]. In this age group, recurrent abdominal pain could be a symptom of recurrent UTI and should be evaluated promptly.

In older children, fever is usually the presenting symptom of UTI. A fever of greater than 38°C without a source has a positive likelihood ratio of 3.6 and with temperatures greater than 39°C have a positive likelihood ratio of 4 [11]. Besides fever, children may have vomiting, loose stools, and abdominal pain [17]. This age group could present with more specific symptoms of either cystitis or pyelonephritis. These may include dysuria, frequency, new onset incontinence flank pain, and fever. Sometimes, however, younger children may have short periods of urgency not associated with UTI. 

Adolescent girls may have urethritis from an STD. Hence, for proper diagnosis, laboratory evaluation is mandatory [5].

The recurrence rate for UTI is 12% after a first time UTI [10].

### 5.2. Lab Investigation

#### 5.2.1. Urine Culture

Urine in the bladder is usually sterile; thus any bacteria growing in should be considered an infection. Pryles reviewed the existing pediatric data in 1960 defined UTI in children [22]. This definition is still valid today. He stated that urine cultures with fewer than 10^3^ colony-forming units per mL were almost always contamination, those with between 10^4^ and 10^5^ colony-forming units per mL were suspicious and should be repeated, and those with more than 10^5^ colony-forming units per mL were indicative of infection [3].

Unfortunately, oftentimes the culture will grow a bacterium that is obviously a contaminant, either from the skin or from other parts of the genital tract. Such culture often has multiple organisms and colony count less than 10^5^. Thus, most investigators define a UTI as the presence of single organism in the urine combined with signs or symptoms of UTI in the patient [3, 23, 24].

The traditional cutoff for urine obtained by noninvasive collection methods (bag or clean catch) has been 10^5^ CFU/mL [5]. For suprapubic aspiration, 10^2^ CFU/mL is regarded as the cut off [5, 25]. Some people have used 50,000 CFU/mL from catheterized sample [26–28]. 

When there are multiple organisms, or low colony count, there is a higher chance of contamination [29].

#### 5.2.2. Obtaining a Urine Sample

Culture of the urine remains the gold standard for diagnosing UTIs [3, 15]. The significance of bacterial growth from a urine sample depends largely on the method by which urine is obtained and the number of colonies harvested. The culture results from a bagged urine specimen have are only helpful if negative [30, 31]. Hence, a positive urine culture from a bagged specimen cannot diagnose UTI. Suprapubic specimen remains the gold standard [27]. This method is difficult to exercise beyond infancy. Transurethral catheterization is preferred in older children. Catheterization of the urethra is occasionally difficult in patients with phimosis or labial adhesions. Also, the contamination chances although small are still higher than suprapubic aspiration. Significant bacterial (>10^5^) colony count is highly suggestive of UTI. 

As children get older and become toilet trained, mid-stream clean catch sample of urine is commonly used [32, 33]. The contamination rates are within limits if obtained the urethral area is cleansed with soap and water. With improper cleaning, the incidence of contamination increases by three folds [32]. Again, the value of this method is in ruling out rather than diagnosing UTI.

#### 5.2.3. Urine Dipstick

Urine dipstick is helpful for rapid screening till the culture result comes back. The dipstick gives information about nitrites and leukocyte esterase (LE). Nitrites are generated from the breakdown of dietary nitrate by bacteria [34] and leukocyte esterase is the breakdown product of white cells. 

LE alone has a positive predictive value of about 35.8% meaning that it has a false-positive rate of about 64.7% [35]. Nitrites on the other hand, when present, are highly suggestive of UTI. Their absence does not rule out an infection as not all organisms produce nitrites (e.g., Gram-positive and Acinatobacter spp.). Nitrites may not be of significance in infants and small children as the conversion requires 3-4 hours and these children urinate much more frequently [36, 37].

#### 5.2.4. Urine Microscopy

Definition of pyuria is not clear in the literature. Multiple studies and a few meta-analyses [36–38] found the cutoff of 5 WBC per HPF being used, the sensitivity being 74% and specificity being 86%. 

#### 5.2.5. Blood Tests

When the child appears sick, a CBC, CRP, blood culture, and procalcitonin should be obtained to evaluate for sepsis. The first two do not have reliability in differentiating upper from lower urinary tract infection [39]. Blood culture is usually done for sick-looking children and younger infants. About a tenth of young infants have bacteremia with UTI [40]. Bacteremia usually clears within 24 hours with appropriate antibiotics, regardless, or route [5, 13]. Procalcitonin, a proinflammatory marker, is newer and promising but further studies are needed [5, 7, 41].

In infants younger than 8 weeks, lumbar puncture is still recommended as there is lack of evidence to omit this step. There is usually CSF pleocytosis, although meningitis and UTIs are rare together [42].

#### 5.2.6. Imaging

All males and females with well-documented UTIs should be imaged for the presence of urological anomalies associated with UTI. The extent of evaluation varies depending on the age of presentation with the first UTI and severity of the episode. The younger the child, the higher the likelihood of anatomical abnormality, hence all children younger than 2 years. of age with well-documented UTI should be evaluated with a renal ultrasound. Beyond 8 yrs of age, boys with UTIs still warrant a renal ultrasound. Girls with a first time simple UTI can likely be observed [27].

#### 5.2.7. Renal Ultrasound

Renal ultrasound is helpful in delineating anatomic abnormalities [43]. It can also be helpful in detecting renal abscesses and stones [44]. For infants younger than 6 months with first-time UTI that responds to treatment, ultrasound should be carried out within 6 weeks of the UTI. A normal ultrasound does rule out hydronephrosis which when present can suggest either vesicoureteral reflux or obstruction of the urinary tract. 

#### 5.2.8. DMSA (Dimercaptosuccinic Acid) Renal Scan

 A DMSA is a nuclear scan that is often used either to diagnose pyelonephritis or permanent renal scars [9, 45]. During an acute UTI DMSA shows photopenic areas in the kidney. These lesions are either permanent (scars) or represent focal area of infection that eventually resolve. DMSA scan may be needed in 6 months to confirm scarring [46].

#### 5.2.9. Voiding Cystourethrogram (VCUG)

All vesicoureteric reflux is diagnosed by VCUG. VCUG does not need to be performed for every febrile UTI. It should, however, be performed if renal ultrasound shows hydronephrosis or any other sign of VUR [27].

It requires catheterization. The radiation exposure can be reduced by performing a radionucleotide cytourethrogram but this study does not help detect anatomical abnormalities and only grades the reflux into mild–moderate and severe [44]. We use contrast VCUG as the first study for male. Nuclear VCUG is used in all females with UTI and for followup of positive contrast VCUG in females. 

## 6. Management

### 6.1. Acute Treatment

The goal of the acute treatment is to decrease morbidity, and to prevent long-term renal damage. Depending on patient's clinical symptoms and tolerance, therapy can be oral or parenteral as they have both been found equally efficacious. If intravenous antibiotics are used, they can usually be changed to oral in 24 to 48 hours. Parenteral administration of an antimicrobial agent also should be considered when adherence to oral regimen is uncertain [27].

The usual antibiotic choices are cephalosporins, amoxicillin plus clavulanic acid, or trimethoprim sulfamethoxazole. It is also important to be aware local pathogens and antibiotic susceptibility [27]. The total duration of therapy should be 7–14 days [47]. Recurrence rate is high with antibiotic regimen administered for shorter than 7 days [48].

Asymptomatic bacteriuria in infants and children should not be treated with antibiotics [47]. Studies have shown that it disappears over time [12].

### 6.2. Long-Term Management

#### 6.2.1. Bowel and Voiding Habits

Dysfunctional voiding syndromes and constipation should be considered in young children and adolescents with UTI. Symptoms include recurrent UTI, constipation, encopresis, and day-time enuresis. Dysfunctional voiding if unrecognized and not managed properly could lead to reflux nephropathy. This later syndrome is associated with renal scars, hypertension, and chronic kidney disease. Children should be encouraged to void frequently and hydrate well. Children should have ready access to clean toilets when required and should not be expected to delay voiding [47]. We often start prophylactic antibiotics for at least 6 months or until proper voiding habits are regained. There have been no trials to support this practice.

#### 6.2.2. Antibiotic Prophylaxis

In the recent years, the rule of vesicoureteric reflux in UTIs and the role of prophylactic antibiotics in preventing UTIs have been controversial. There have been a few trials in younger children that found no benefit of antibiotic prophylaxis [49, 50]. Antibiotic prophylaxis may be considered in infants and children with recurrent UTI [27]. If needed, the common antimicrobials used are trimethoprim sulfamethoxazole, trimethoprim, nitrofurantoin, and first generation cephalosporins in a one nightly dose. In children less than two months of age, amoxicillin is generally used as prophylaxis [44].

#### 6.2.3. Surgical Treatment of VUR

VUR often undergoes spontaneous resolution. The time from first UTI to resolution of VUR is 6-7 yrs. Comparison of medical and surgical treatment of VUR is hard as different studies use various outcomes. Hodson et al. [51] reported decreased febrile UTIs as the only benefit of surgical management. There was no difference in renal scars or UTIs in general [51]. Surgical treatment for vesicoureteric reflux is reserved for patients with high grade and unilateral reflux, recurrent UTIs despite antibiotic prophylaxis, and noncompliance with antibiotics persistence beyond 9 yrs of age [44]. Endoscopic management involves subureteral or intraureteral injection of bulking agent with dextranomer/hyaluronic acid is suggested as first line treatment [52].

### 6.3. Long Term Followup

Infants and children with uncomplicated UTIs who do not undergo imaging investigations do not require follow up by a subspecialist. Infants and children who have recurrent UTI or abnormal imaging results should be assessed by a pediatric specialist. Assessment of infants and children with renal parenchymal defects should include height, weight, blood pressure, and routine testing for proteinuria. Infants and children with a minor, unilateral renal parenchymal defect do not need long-term followup unless they have recurrent UTI or family history or lifestyle risk factors for hypertension [47].

Infants and children who have bilateral renal abnormalities, impaired kidney function, raised blood pressure, and/or proteinuria should receive monitoring and appropriate management by a pediatric nephrologist to slow the progression of chronic kidney disease.

Infants and children who are asymptomatic following an episode of UTI should not routinely have their urine re-tested for infection. Asymptomatic bacteriuria is not an indication for followup [47].

### 6.4. Parent Education

Healthcare professionals should ensure that when a child or young person has been identified as having a suspected UTI, they and their parents are given information about the need for treatment, the importance of completing any course of treatment and advice about prevention and possible long-term management [47].

Parents should be made aware of the possibility of a UTI recurring and understand the need to be vigilant and to seek prompt treatment from a healthcare professional for any suspected reinfection.

Parents should be educated about healthy voiding and stooling habits as means of preventing UTIs.

## 7. Summary: The Disease from a GP Perspective

Urinary tract infections are common in children. If recurrent or severe, they do have the potential to cause renal scarring. All younger infants with fever of unexplained origin should have their urine tested and older children with symptoms should also be evaluated for UTIs. The gold standard for testing for UTI is suprapubic aspiration but a urinalysis and a urine culture (catheterized/clean catch depending on age) is acceptable. Once diagnosed, prompt and appropriate antibiotic treatment can prevent long-term complications and scarring. All younger infants with UTI and older children with complicated UTI should get a renal ultrasound. This should be followed by VCUG only if there is evidence of reflux on ultrasound. A DMSA scan can help evaluate renal scarring. Prophylactic antibiotics are reserved for recurrent UTIs and do not seem to benefit patients with low-grade VUR. Preventative measures include treating constipation and voiding dysfunction.
